# Supplementary material for: A chemiresistive-potentiometric multivariate sensor for discriminative gas detection
Source: Nat Commun. 2023 Jun 13;14:3495. doi: 10.1038/s41467-023-39213-x (PMC10264437; doi:10.1038/s41467-023-39213-x)
Supplement: Supplementary file 1 — Supplementary Information File [file 41467_2023_39213_MOESM1_ESM.pdf]

Supplementary Information for

A chemiresistive-potentiometric multivariate sensor for discriminative gas detection

*Hong Zhang, Zuobin Zhang, Zhou Li, Hongjie Han, Weiguo Song, Jianxin Yi**

State Key Laboratory of Fire Science, Department of Safety Science and Engineering, University of Science and Technology of China, Hefei, Anhui, 230026, P.R. China

* *Corresponding Author. E-mail address: yjx@ustc.edu.cn.*

Contents

Supplementary Notes 2

Supplementary Figures 5

Supplementary Tables 35

Supplementary References 40

Supplementary Notes

Supplementary Note 1

Synthesis of NiO, Fe-doped NiO, and ZnO nanofibers

All chemicals were purchased from Sinopharm Chemical Reagent Co., Ltd, China, unless stated otherwise. NiO NFs and Fe-doped NiO NFs were prepared via electrospinning. In a typical process, homogenous Ni-source solution was obtained by dissolving 1 g $Ni(NO_3)_2 \cdot 6H_2O$ (≥98%) and 2 g Polyvinyl pyrrolidone (PVP, MW=1.3×10^6, Alfa Aesar) into 8.5 g N,N-dimethylformamide (DMF, ≥ 99.5%) and 8.5 g anhydrous ethanol (≥99.7%) under stirring. 0.00695, 0.0417, 0.1389 g of $Fe(NO_3)_3 \cdot 9H_2O$ (≥98.5%) were added to the aforementioned Ni-source solution in order to prepare 0.5, 3, and 10 at.% Fe-doped NiO NFs, respectively. The Ni-source solution with or without an Fe source was homogenized for 24 h and then transferred to a plastic syringe mounted on a syringe pump (LSP01-1A). Electrospinning was conducted at a voltage of 18 kV at an injection rate of 0.4 ml/h and a working distance of 20 cm. The as-spun nanofiber mats dried at 80°C and calcined at 600°C for 3 h to obtain NiO NFs and Fe-doped NiO NFs (Pristine NiO NFs and Fe-doped NiO NFs with nominal Fe/Ni ratio of 0.5, 3, and 10 at.% were denoted as NiO-0, NiO-0.5, NiO-3, and NiO-10, respectively).

ZnO NFs were prepared in a similar procedure. Typically, homogenous electrospinning precursor was obtained by dissolving 0.7 g $Zn(NO_3)_2 \cdot 6H_2O$ (≥99%) and 0.8 g PVP into 4.7 ml DMF and 5.6 ml anhydrous ethanol under stirring. Electrospinning was conducted at a voltage of 18 kV at an injection rate of 0.4 ml/h and a working distance of 20 cm. The as-spun nanofiber mats dried at 80°C and calcined at 500°C for 3 h to obtain ZnO NFs.

Supplementary Note 2

Fabrication of ESB and NASICON solid electrolytes

ESB powders were synthesized by a sol-gel method. Stoichiometric amounts of Er_2O_3 (≥99.9%) and $Bi(NO_3)_3$ $6H_2O$ (≥99%) were dissolved in nitric acid solution to form nitrate solution. EDTA (⩾ 99.5%) were dissolved in distilled water, citric acid (≥99.5%) and $NH_3{\cdot}H_2O$ was added to form complexing agent. The mole ratio of EDTA, citric acid, and total metal ions= 1:2:1. The nitrate solution was slowly dropped into the complexing agent under stirring until the PH of the mixed solution to 7. The mixed solution was evaporated to dry gel under continuous stirring and heating, and the dry gel was calcined at 800 ℃ for 5 h to obtain ESB powders. 0.4 g ESB powders were pressed into a disk at a uniaxial pressure of 218 MPa. After sintering the disk at 800 ℃ for 10 h, ESB ceramic disks of 14.0 mm diameter and 0.5 mm thickness were obtained.

NASICON powders were prepared by a solid-state reaction method. Appropriate amounts of Na_2CO_3 (≥99.8%), $NH_4H_2PO_4$ (≥99%), SiO_2 (≥99.8%), and ZrO_2 (≥99%), with 10 mol% rich of sodium to compensate for Na loss during the following calcination and sintering process, were mixed well in ethanol by ball milling and then dried at 80 ℃ in air. The obtained powder mixture was uniaxially pressed into pellets. Then, the pellets were buried in their unmolded powder mixture (mother powder), and calcined at 1100 ℃ for 10 h in air. The calcined pellets were crashed and ball-milled again to prepare the NASICON powders. The NASICON powder of 0.4 g were pressed into a disk at a uniaxial pressure of 180 MPa. After sintering the disk at 1220 ℃ for 10 h, NASICON ceramic disks of 13.5 mm diameter and 1 mm thickness were obtained.

Supplementary Note 3

Characterization and gas sensing tests

Phase structure of the sensing materials and electrolytes was identified by X-ray powder diffraction (XRD, Rigaku TTR-III) using Cu Kα radiation. The morphologies and microstructures were investigated by scanning electron microscopy (FE-SEM, JEOL JSM-6700F) operated at an accelerating voltage of 20 kV.

The sensing characteristics of multivariable sensor were measured using a home-built sensor test system. Each sample gas was obtained by mixing dry air and a stream of certified standard gas of ethanol (7×10^{-3}mol/mol), acetone (6×10^{-3}mol/mol), toluene (2×10^{-4}mol/mol), H_2 (1×10^{-2}mol/mol), NH_3 (1×10^{-2}mol/mol), CO (2×10^{-3}mol/mol), and NO_2 (1×10^{-2}mol/mol) purchased from Nanjing Special Gas Co., Ltd. 2-EH vapors were obtained by bubbling dry air through a glass bubbler containing 2-EH (⩾99.6%, Sigma-Aldrich), and the mixture was subsequently diluted with air again to adjust the concentration of 2-EH. Gas flow rates were adjusted by the mass flow controllers (MFC, CS200, Beijing Sevenstar Electronics, China), and the total flow rate was fixed at 200 ml/min (200-250 ml/min for toluene). To investigate the effect of relative humidity (RH) on sensing performance, dry air was bubbled through saturated solution of CH_3COOK (21.3% RH) or K_2CO_3 (49.8% RH), or deionized water (96.5% RH) and subsequently mixed with the analyte gas stream. The RH values were measured using a humidity sensor (TESTO, 605-H1).

To evaluate the sensing characteristics of the multivariable sensor to fire hazards, a cylindrical container (3.5 cm×6 cm) with a ceramic heating plate (XH-RP1010, Jiangsu, 1 cm×1 cm) was installed ahead of the sensor chamber. Fuel samples of Cable 1(BV 2.5, Yuandong), Cable 2 (BV 2.5, Gamma), Cotton and Beech were ultrasonically cleaned in water before use, and samples of paper were used as received. 0.1 g fuel was placed and fixed at the center of the heating plate, whose heating temperature was controlled by a DC power supply (Querli, Shanghai) and monitored with a thermocouple (Omega, USA). The fuel vapors at different overheating temperature were carried by an air stream of 200 ml/min into the sensor chamber in real time. For comparison, a Honeywell photoelectric smoke detector (JTYJ-GD-01LM/BW) was also employed to detect the fuel vapors under the same operating conditions.

Supplementary Figures

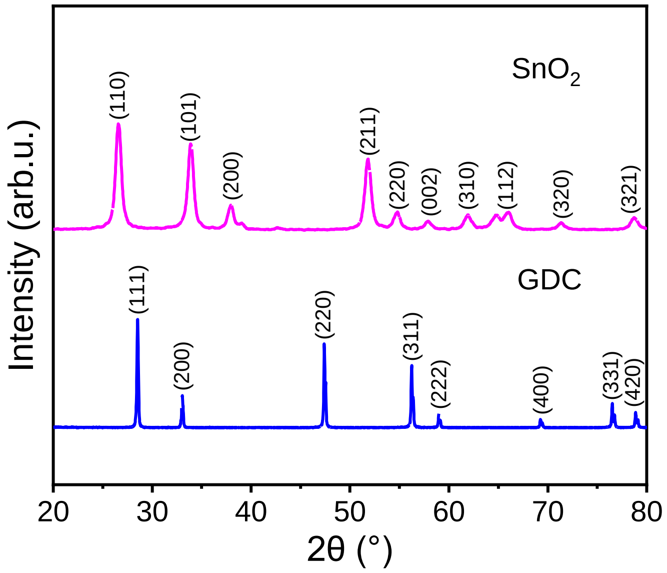

Supplementary Fig. 1. XRD pattern of as-synthesized SnO_2 nanofibers and GDC powders.

As shown in Supplementary Fig. 1, the crystal structures of SnO_2 nanofibers (NFs) and GDC electrolyte are well indexed as single-phase tetragonal rutile structure (JCPDS No.: 41-1445) and fluorite structure (JCPDS No.: 50-0201), respectively.

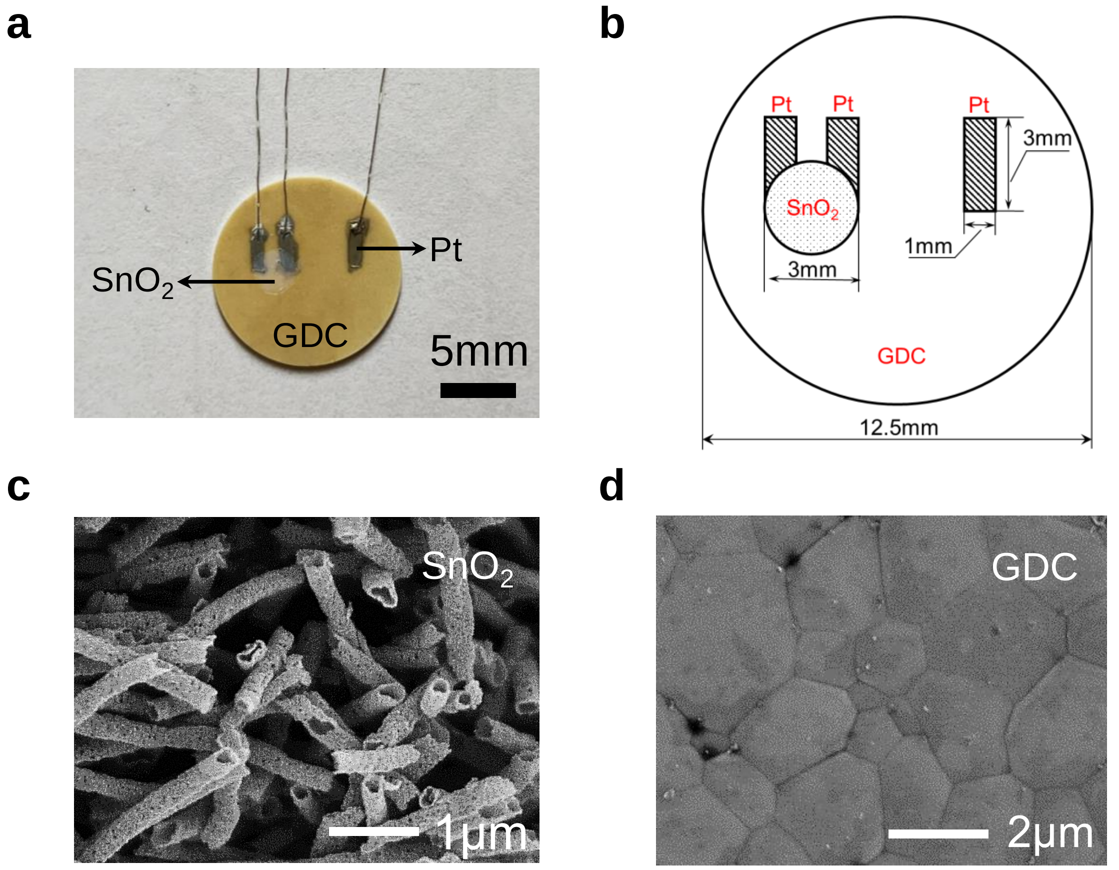

Supplementary Fig. 2. (a) Photograph and (b) schematic of the SnO_2 C-P sensor; surface SEM image of (c) SnO_2 nanofibers SE and (d) GDC electrolyte disk.

As shown in Supplementary Fig. 2a-b, striped Pt current collectors and Pt CE with an effective area of 1 mm×3 mm were made on a GDC electrolyte disk with a diameter of 12.5 mm. Pt wires were connected to the top of the striped Pt current collectors and Pt CE with small point-shaped Pt dots. Subsequently a circular SnO_2 SE with an effective diameter of 3 mm was coated on the bottom of the Pt collectors to form the SnO_2 C-P sensor. The size of C-P sensor can be significantly reduced by improving the fabrication process.

Supplementary Fig. 2c displays that the SnO_2 NFs had a hollow core and a uniform outer diameter of ~230 nm. The highly porous hollow NFs are randomly but evenly stacked upon each other in the sintered NFs SE, establishing a three-dimensional scaffold architecture with a high degree of (sub)micron-sized void space among them. As shown in Supplementary Fig. 2d, GDC electrolyte disk is dense with typical grain size of 780 nm-3.6 μm after sintered at 1500 ℃.

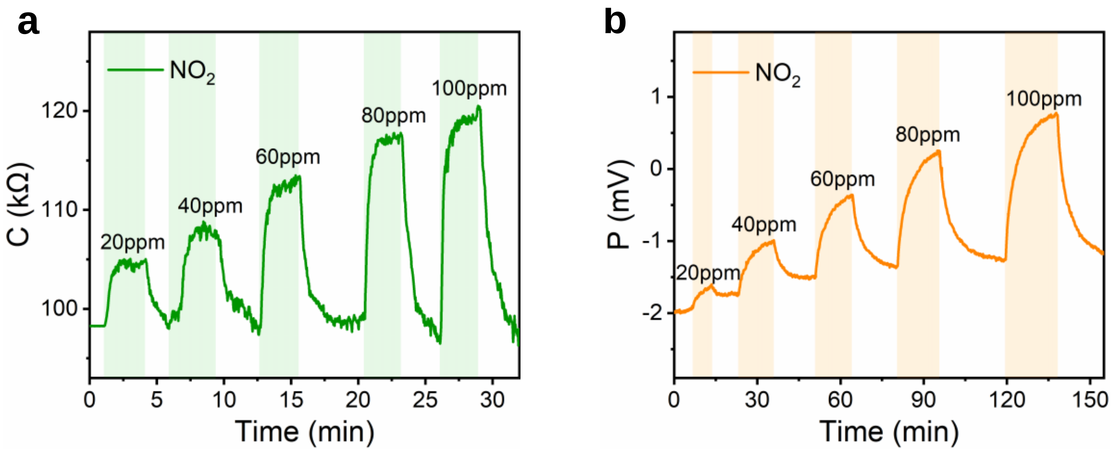

Supplementary Fig. 3. (a) Chemiresistive and (b) potentiometric response and recovery transients of the multivariable sensor based on SnO_2 SE in different concentrations of NO_2 at 400 ℃.

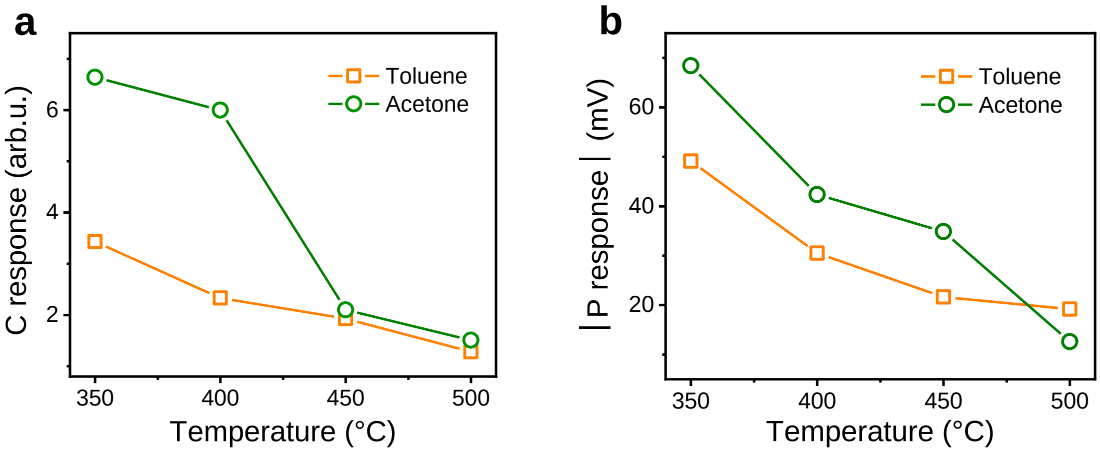

Supplementary Fig. 4. (a) Chemiresistive and (b) potentiometric responses of SnO_2 toward 100 ppm toluene and acetone as a function of operating temperature.

Supplementary Fig. 4 shows the temperature dependence of the chemiresistive and potentiometric responses to 100 ppm of toluene and acetone for SnO_2 NFs. The response values for both signals significantly increase with decreasing operating temperature. Moreover, the operating temperature of chemiresistive and potentiometric sensing matches well with each other, and the multivariable sensing of SnO_2 NFs can work in a wide working temperature range of 350-500 ℃.

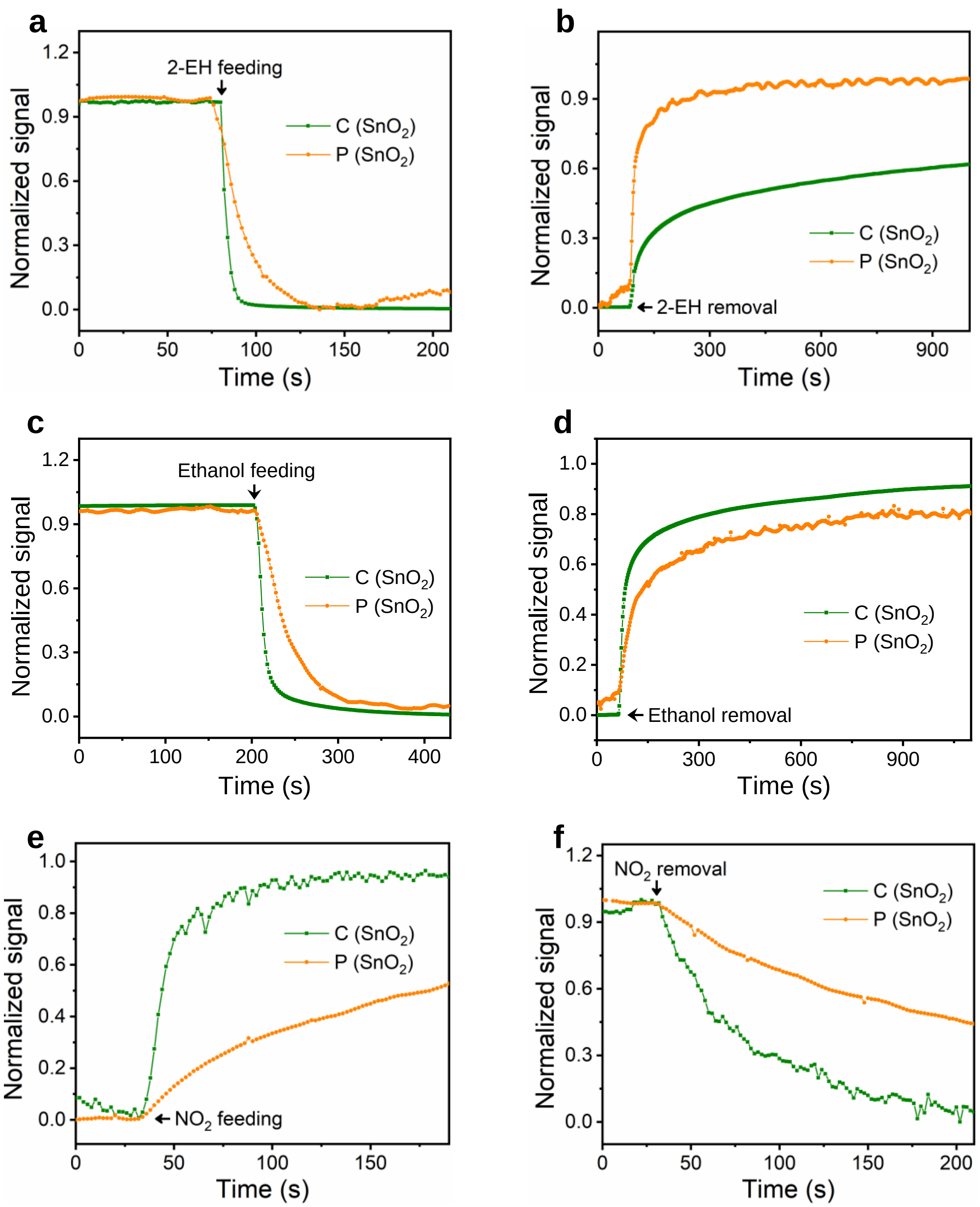

Supplementary Fig. 5. Normalized chemiresistive and potentiometric (a, c, e) response and (b, d, f) recovery curves of SnO_2 nanofibers to 100 ppm (a-b) 2-EH, (c-d) ethanol and (e-f) NO_2 at 400 ℃.

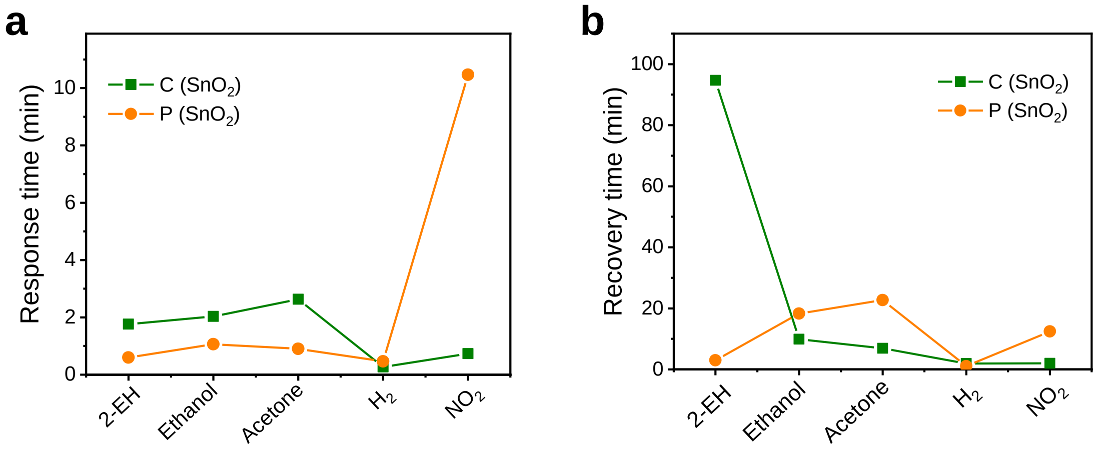

Supplementary Fig. 6. Chemiresistive and potentiometric (a) response time and (b) recovery time of SnO_2 nanofibers toward 5 common gases of 100 ppm at 400 ℃.

Supplementary Fig. 5-6 show that the chemiresistive and potentiometric signals differ significantly in the dynamic curves and response/recovery time, which agrees well with their different mechanisms, suggesting independence of the two signals.

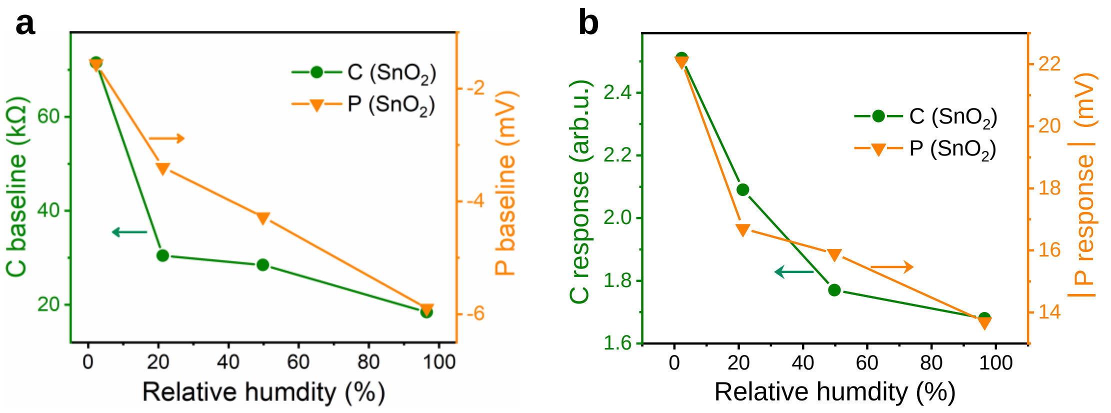

Supplementary Fig. 7. (a) Baseline and (b) response values to 100 ppm toluene for SnO_2 at 400 ℃ under different relative humidity.

As shown in Supplementary Fig. 7, with the relative humidity increasing from 2.3% to 96.5%, the baseline resistance and potential as well as the chemiresistive and potentiometric responses to toluene decrease significantly. These results reveal that the chemiresistive and potentiometric behaviors of SnO_2 are adversely affected by the humidity.

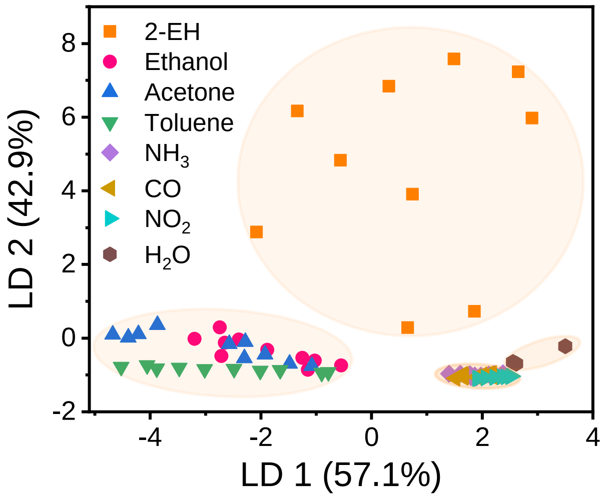

Supplementary Fig. 8. Pattern recognition for 8 target gases in the concentration range of 20-100 ppm based on LDA using the C-P sensor with SnO_2 nanofiber SE.

To quantitatively evaluate the gas discriminative capability of the multivariable sensor with SnO_2 SE, the response values were treated by linear discrimination analysis (LDA), a common supervised statistical analysis technique. Supplementary Fig. 8 shows pattern recognition for eight target gases in the concentration range of 20-100 ppm based on LDA. It can be seen that the eight target gases can be classified into four groups without overlap, i.e., 2-EH, other VOCs, inorganic gases, and humidity, thereby distinguishing at least four gases in eight target gases.

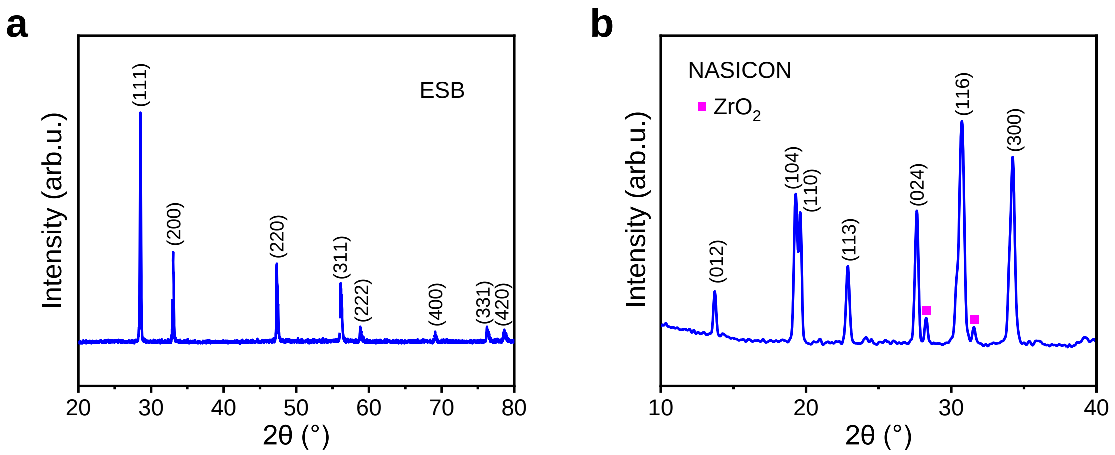

Supplementary Fig. 9. XRD patterns of (a) ESB and (b) NASICON electrolytes.

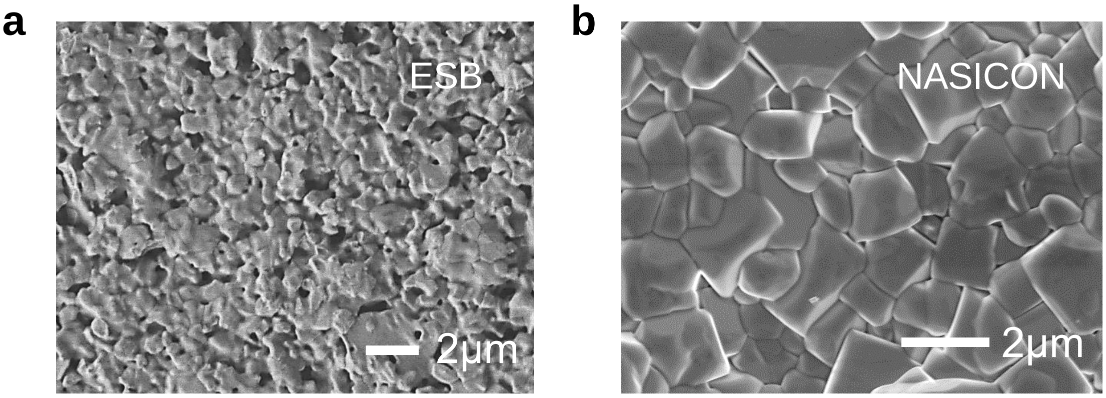

Supplementary Fig. 10. Surface SEM images of (a) ESB and (b) NASICON electrolytes.

As shown in Supplementary Fig. 9a, single phase fluorite structured ESB (JCPDS No.: 34-0377) were obtained with high crystallinity. XRD patterns of NASICON were indexed as a monoclinic structure (JCPDS No.: 33-1314), in which the peaks of ZrO_2 (JCPDS No.: 86-1450) at ~28.28 °and ~31.51 °were observed (Supplementary Fig. 9b). ZrO_2 was a common impurity caused by the volatility of Na and P during high temperature calcination of NASICON. Supplementary Fig. 10 shows surface SEM images of ESB and NASICON solid electrolytes. Dense structure was observed for NASICON, while some small pores appeared for ESB probably due to the relatively low sintering temperature (800 ℃).

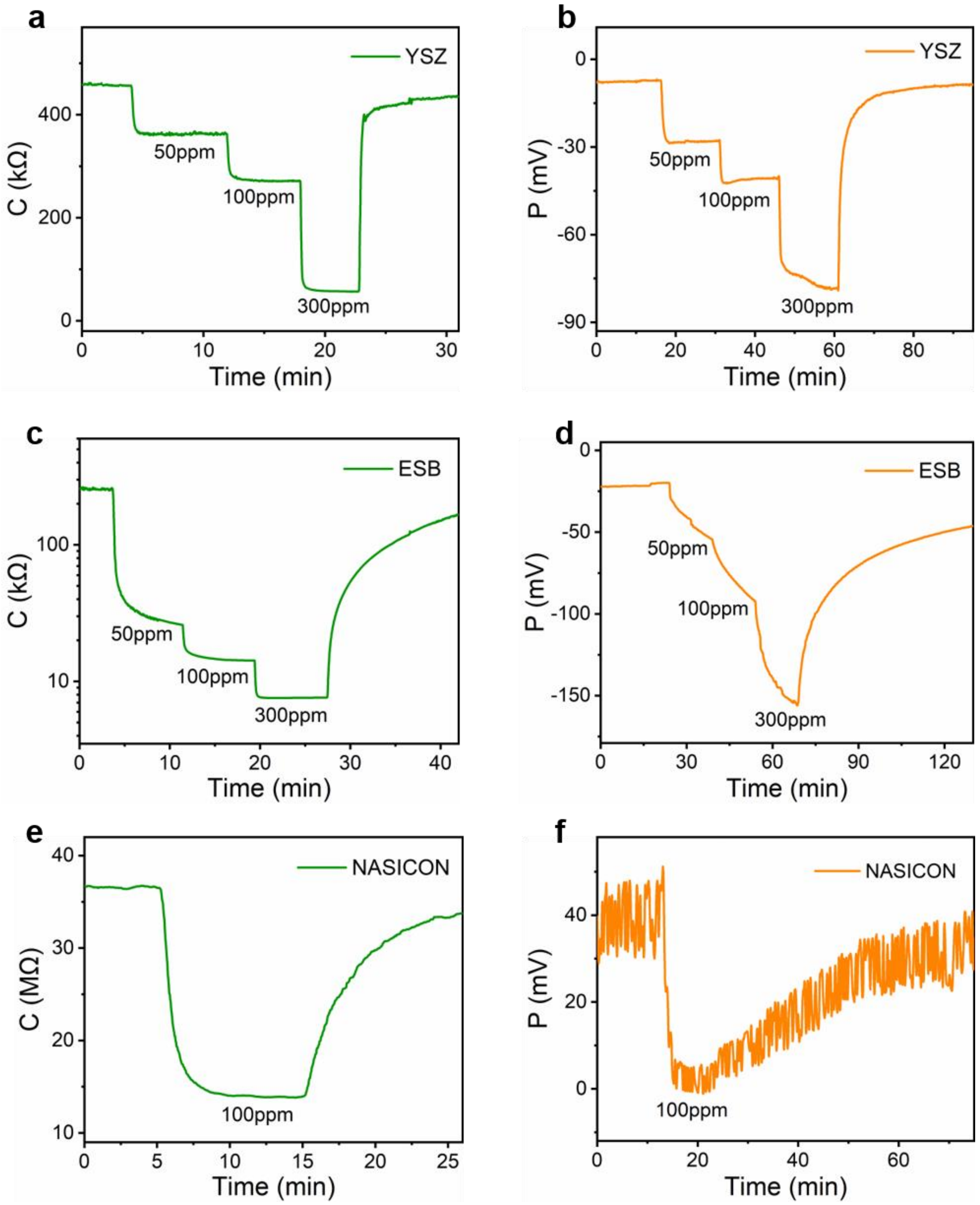

Supplementary Fig. 11. (a, c, e) Chemiresistive and (b, d, f) potentiometric response curves toward ethanol of SnO_2 nanofibers on (a-b) YSZ at 400 ℃, (c-d) ESB at 300 ℃ and (e-f) NASICON at 200 ℃.

Supplementary Fig. 11 shows clearly n-type chemiresistive and non-Nernstian gas sensing behavior of SnO_2 NFs on YSZ, ESB, and NASICON, similar to that on GDC (Fig. 1). Furthermore, the lowest operable temperature of multivariable sensing are around 400 ℃, 300 ℃ and 200 ℃, respectively, which agrees with that higher ion conductivity generally favors lower potentiometric working temperature.

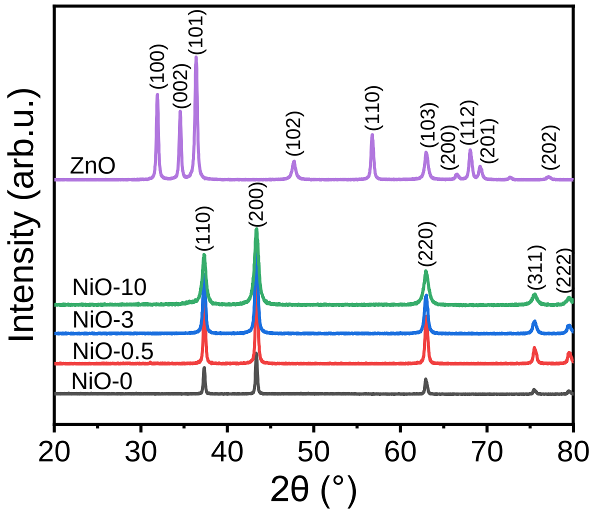

Supplementary Fig. 12. XRD patterns for nanofibers of ZnO, NiO, and Fe-doped NiO.

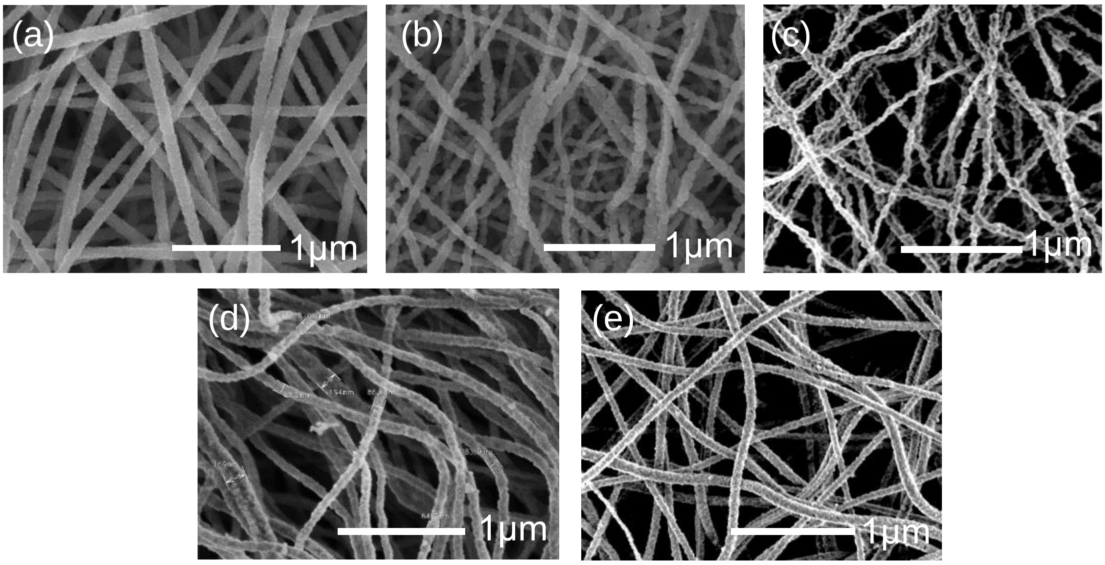

Supplementary Fig. 13. SEM images of nanofibers. (a) ZnO, (b) NiO-0, (c) NiO-0.5, (d) NiO-3, and (e) NiO-10.

Supplementary Fig. 12 shows XRD patterns of ZnO NFs, NiO NFs and three Fe-doped NiO NFs. It can be seen that hexagonal wurtzite structure for ZnO NFs (JCPDS No.: 75-0576) and cubic phase structure for NiO NFs (JCPDS No.: 47-1049) were obtained. For three Fe-doped NiO NFs, no second phase was found when compared to that of the pure NiO NFs. The ionic radii of Fe^{2+} and Fe^{3+} at a coordination number (CN) of 6 are 0.64 Å and 0.74 Å, respectively, which are comparable with that of Ni^{2+} at a CN of 6 (0.69 Å)[1, 2]. Thus, Fe ions could enter the NiO lattice without changing the crystal

structure of NiO. Fe/Ni atomic ratios for NiO-0.5, NiO-3 and NiO-10 were 0.64 %, 2.47 %, and 8.69 %, respectively, determined by EDS analyses.

Supplementary Fig. 13 shows typical SEM images of ZnO NFs, NiO NFs, and three Fe-doped NiO NFs. Nanofiber morphology was observed for the five materials. The ZnO NFs have an outer diameter of 100-120 nm while NiO NFs and Fe-doped NiO NFs have a similar outer diameter of 60-120 nm.

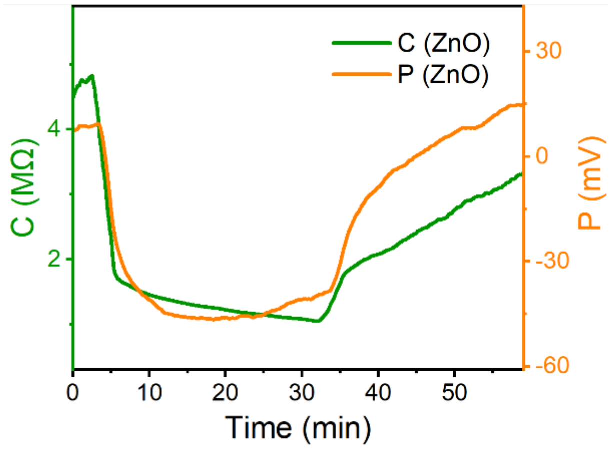

Supplementary Fig. 14. Chemiresistive and potentiometric response curves of ZnO nanofibers on GDC in the presence of 500 ppm acetone at 400 ℃.

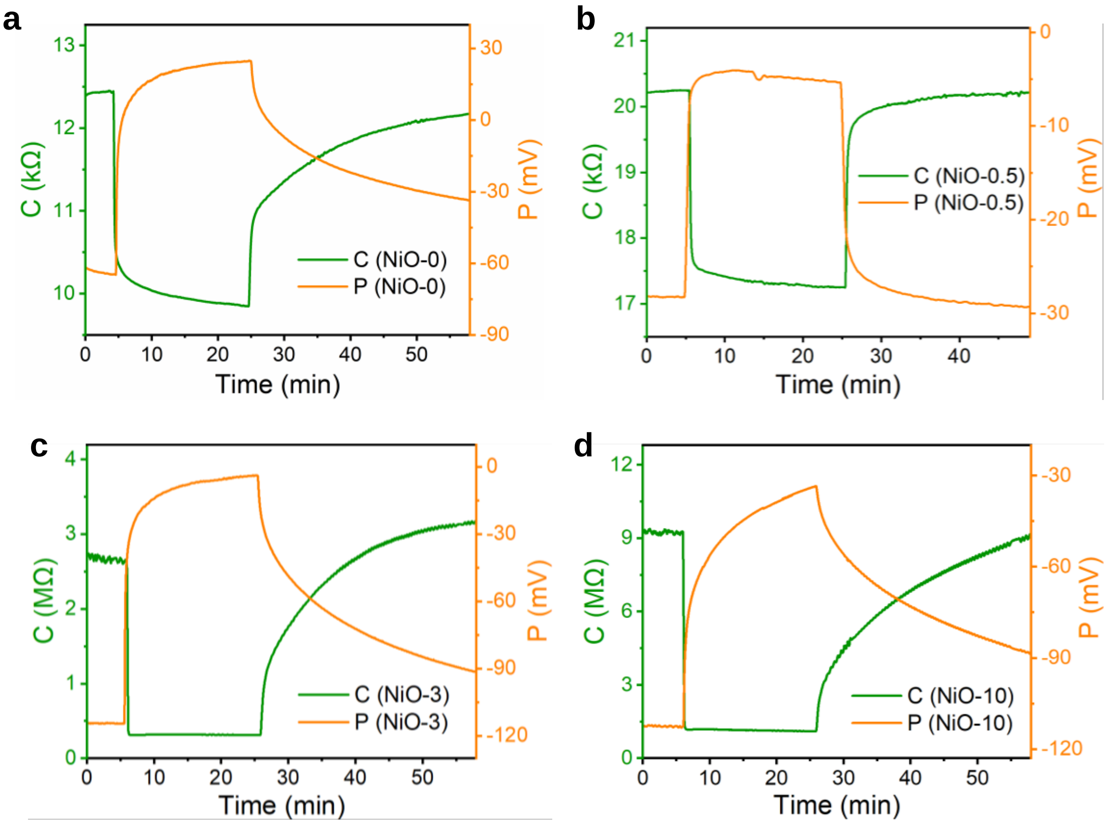

Supplementary Fig. 15. Chemiresistive and potentiometric response curves of (a) NiO-0, (b) NiO-0.5, (c) NiO-3, and (d) NiO-10 nanofibers on GDC in the presence of 500 ppm NO_2 at 400 ℃.

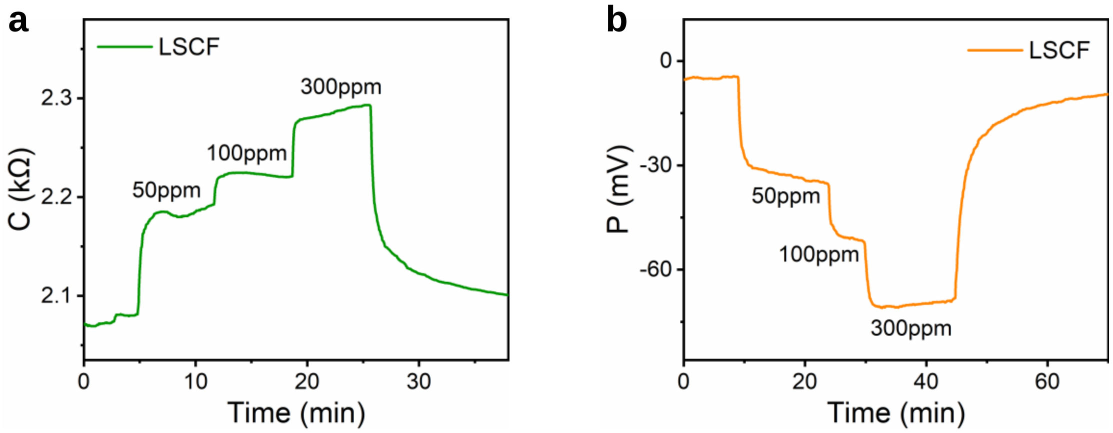

Supplementary Fig. 16. (a) Chemiresistive and (b) potentiometric response curves of LSCF nanoparticles on YSZ in the presence of acetone from 50 to 300 ppm at 400 ℃.

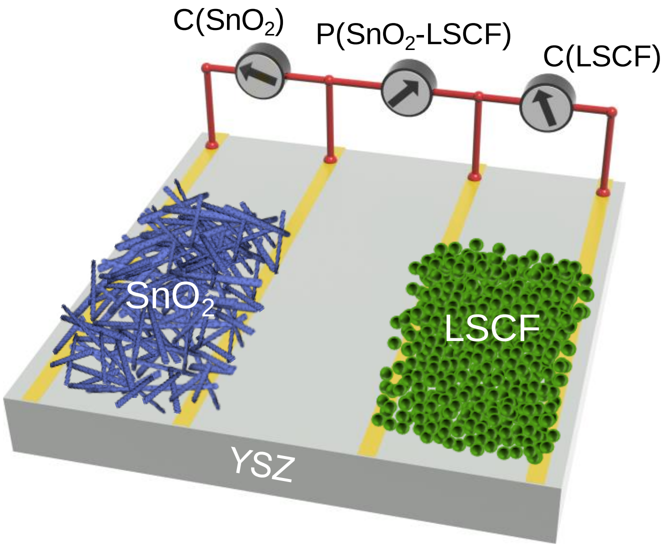

Supplementary Fig. 17. Schematic of the multivariable sensor with SnO_2 and LSCF dual SEs.

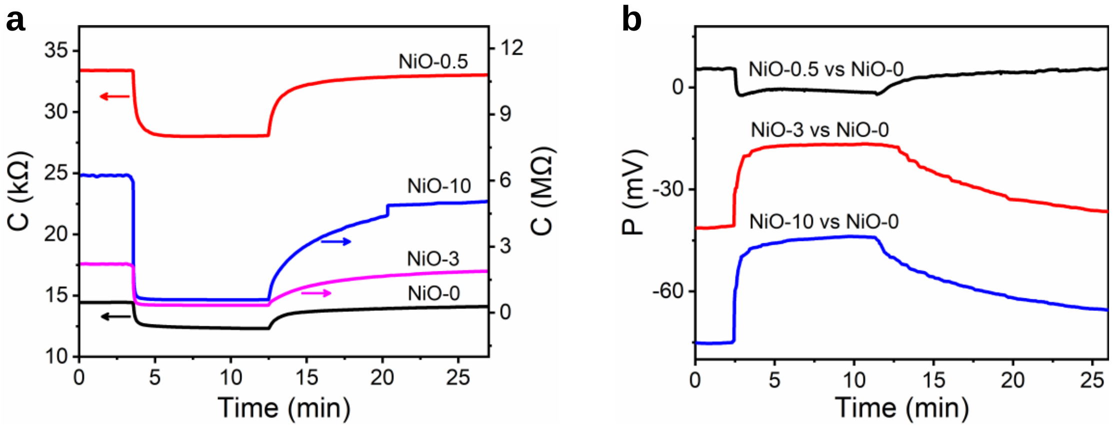

Supplementary Fig. 18. Dynamic (a) chemiresistive and (b) potentiometric response curves of four-SE platform based on NiO-0, NiO-0.5, NiO-3, and NiO-10 SEs in the presence of 500 ppm NO_2 at 350 ℃. Seven independent outputs, four resistance and three potential ones, can be obtained from the four-SE C-P sensor.

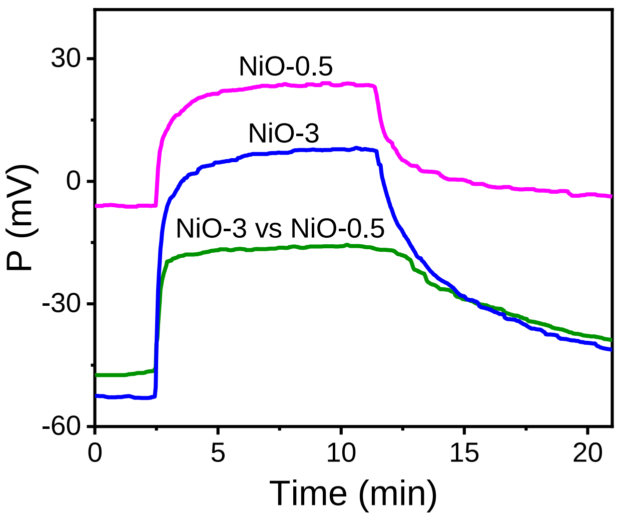

Supplementary Fig. 19. Dynamic potentiometric response curves of the sensor by pairing NiO-3 and NiO-0.5 SEs in the presence of 500 ppm NO_2 at 350 ℃. As responses from different SEs have the same polarity, they partially counteract each other, reducing the P response of the sensor.

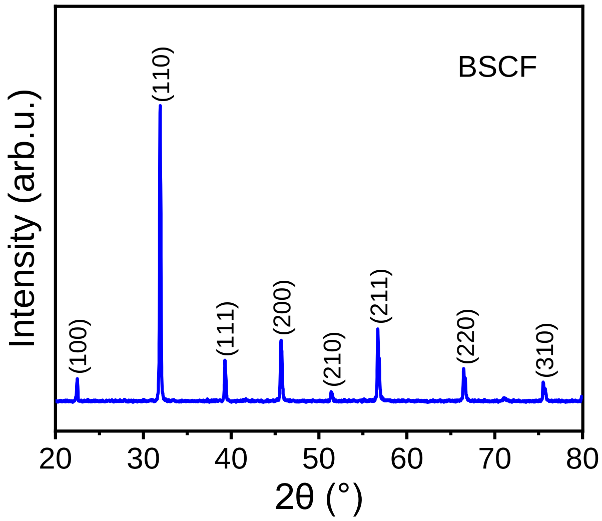

Supplementary Fig. 20. XRD pattern of as-synthesized $Ba_{0.5}Sr_{0.5}Co_{0.8}Fe_{0.2}O_{3-\delta}$ nanoparticles.

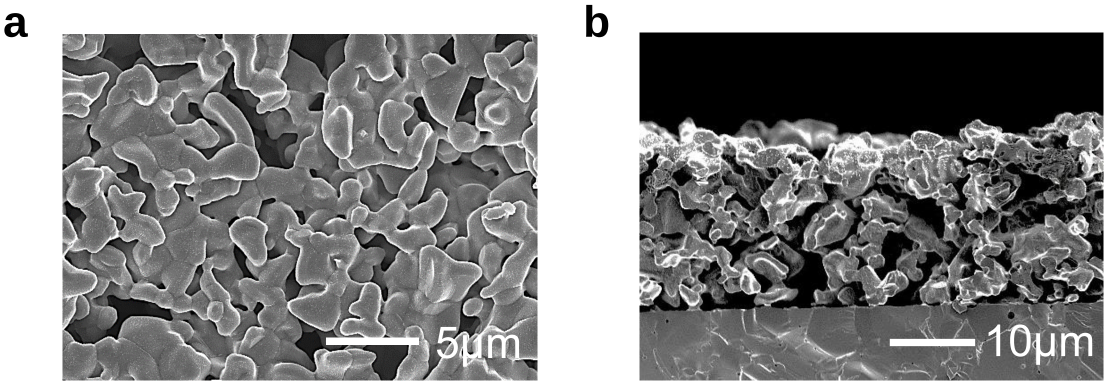

Supplementary Fig. 21. (a) Surface and (b) cross-section SEM images of BSCF SE.

The crystal structure of BSCF can be well indexed as a cubic perovskite structure (JCPDS No.: 039-1083) (Supplementary Fig. 20). SEM images in Supplementary Fig. 21 show that both the BSCF SE are highly porous after sintered at 950 ℃, which would be beneficial to the gas transport during the sensing measurements. The BSCF SE has a uniform thickness of ~20 μm.

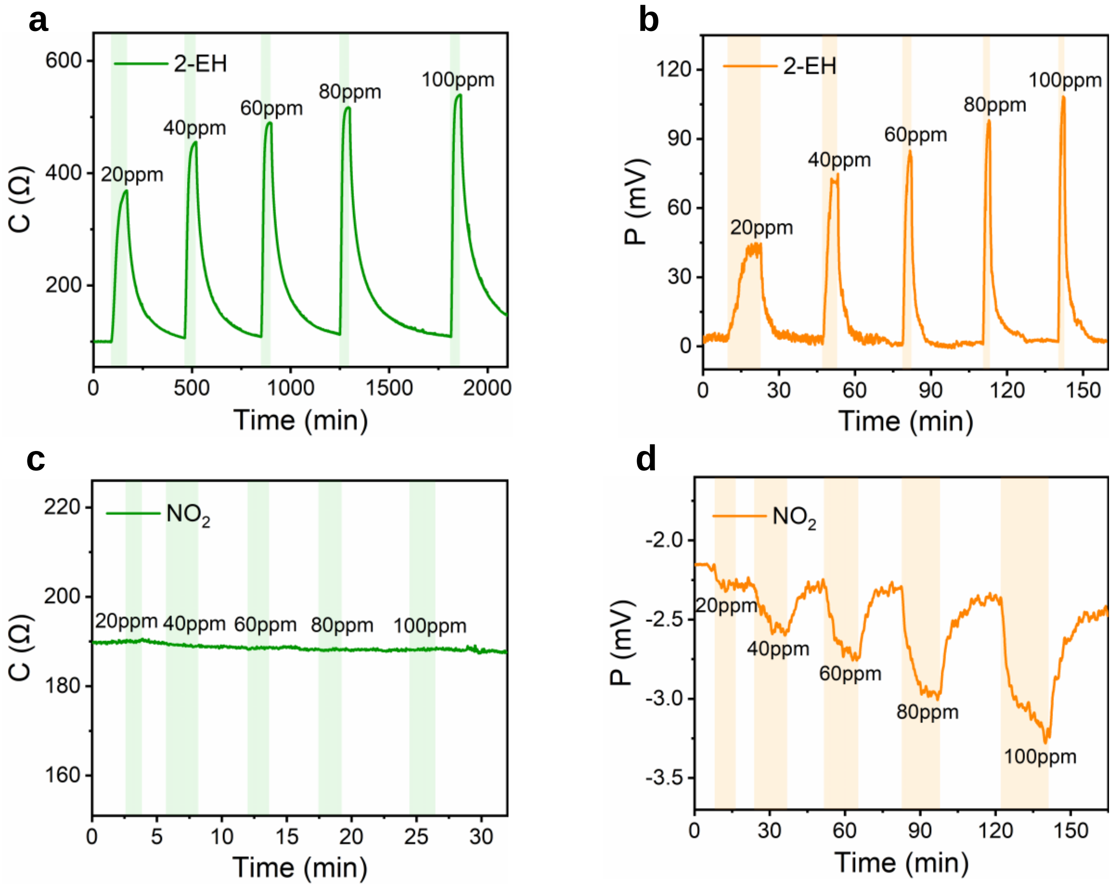

Supplementary Fig. 22. (a, c) Chemiresistive and (b, d) potentiometric response and recovery transients of the multivariable sensor based on BSCF SE in different concentrations of (a-b) 2-EH and (c-d) NO_2 at 400 ℃.

As shown in Supplementary Fig. 22, upon exposure of the device to the reducing (oxidizing) analyte gas, the potential (vs Pt) increases (decreases) substantially. The response polarity is opposite to that of conventional materials. Besides, BSCF exhibits typical p-type chemiresistive behavior, responding sensitively to 2-EH but almost negligibly to NO_2.

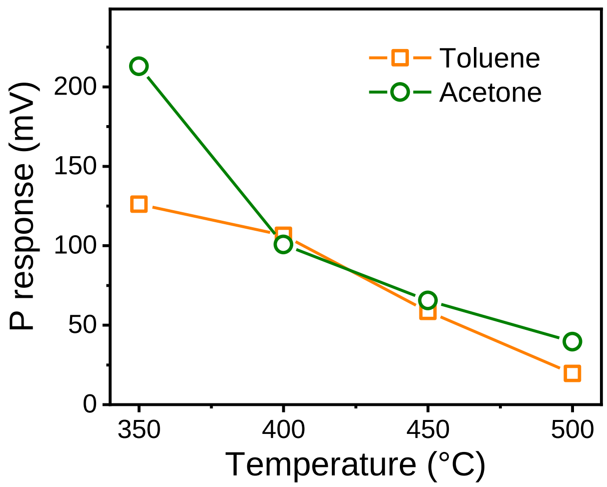

Supplementary Fig. 23. Potentiometric responses of BSCF toward 100 ppm acetone and toluene as a function of the operating temperature.

Supplementary Fig. 23 shows the temperature dependence of the potentiometric response to 100 ppm of toluene and acetone for BSCF. The potentiometric response significantly increases, i.e., becomes more positive, with decreasing operating temperature from 500 ℃ to 350 ℃.

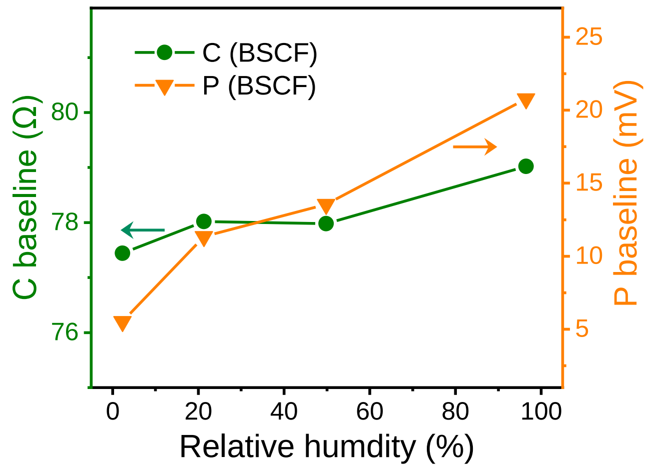

Supplementary Fig. 24. The base resistance and voltage of BSCF under different relative humidity at 400 ℃.

As shown in Supplementary Fig. 24, as the relative humidity increased from 2.3% to 96.5%, the base resistance for BSCF increases only very slightly by ~2%, while the base voltage increases moderately by ~15 mV.

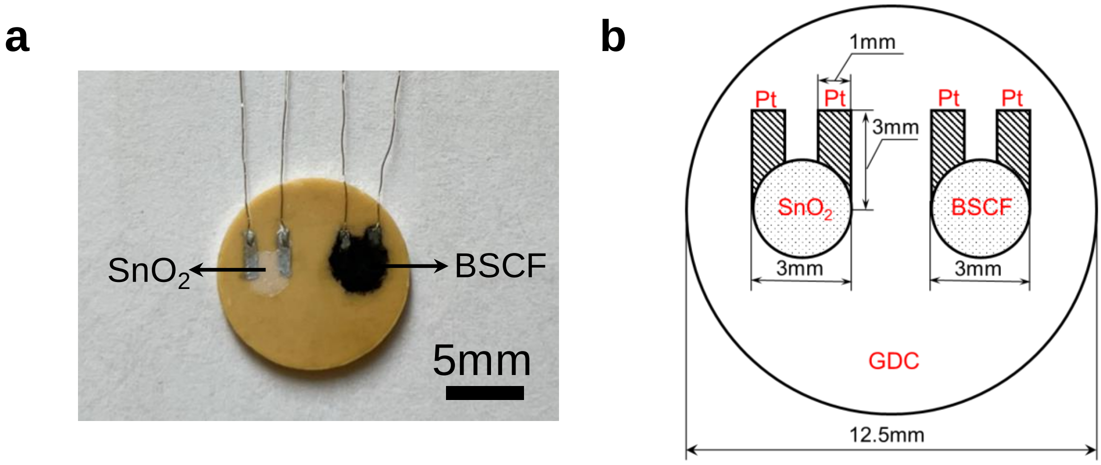

Supplementary Fig. 25. (a) Photograph and (b) schematic of the dual-SE SnO_2-BSCF C-P sensor.

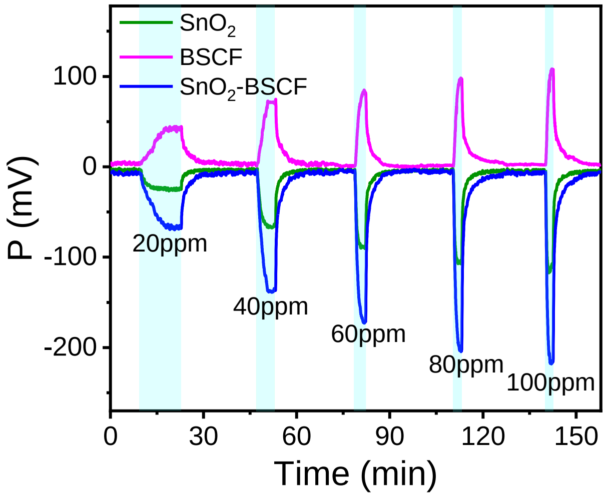

Supplementary Fig. 26. Potentiometric response and recovery transients of SnO_2, BSCF, and SnO_2-BSCF in different concentrations of 2-EH at 400 ℃.

Supplementary Fig. 26 shows potentiometric response and recovery transients of SnO_2, BSCF and SnO_2-BSCF in 2-EH at 400 ℃. Clearly, the potential signal of SnO_2-BSCF is always equivalent to the potential difference of SnO_2 and BSCF, P(SnO_2-BSCF)=P(SnO_2)-P(BSCF). Furthermore, the potentiometric response and sensitivity of the SnO_2-BSCF sensor equals the sum of those for the two single-SE sensors (all in absolute values) due to the opposite potential polarities of SnO_2 and BSCF.

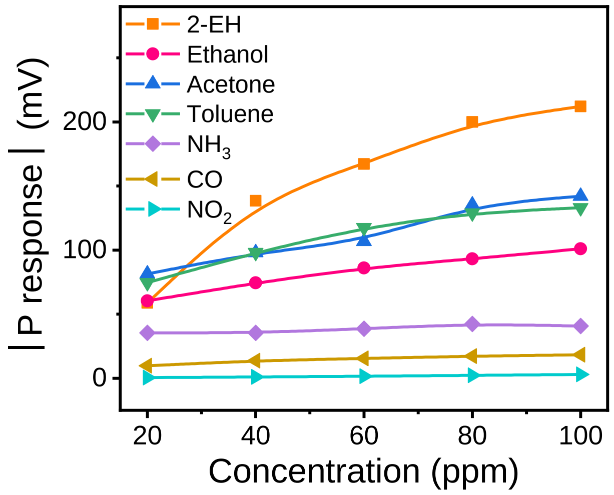

Supplementary Fig. 27. Potentiometric response values of SnO_2-BSCF sensor as a function of gas concentration at 400 ℃.

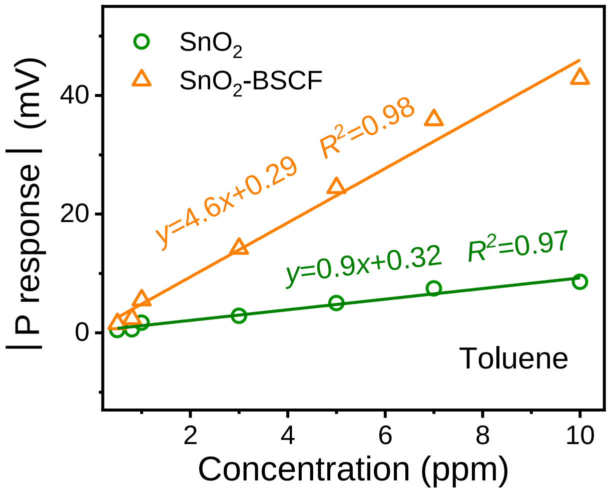

Supplementary Fig. 28. Linear relationships between potentiometric response values and toluene concentration for the SnO_2 and SnO_2-BSCF sensors at 400 ℃.

Supplementary Fig. 28 presents the linear relationships between potentiometric response values and toluene concentration for the SnO_2 and SnO_2-BSCF sensors. Compared with the toluene sensitivity of SnO_2 sensor (0.9 mV/ppm), SnO_2-BSCF has a larger value of 4.6 mV/ppm, corresponding to an increase of 411%. The limit of detection of toluene is calculated for the SnO_2-BSCF sensor to be as low as 55.9 ppb.

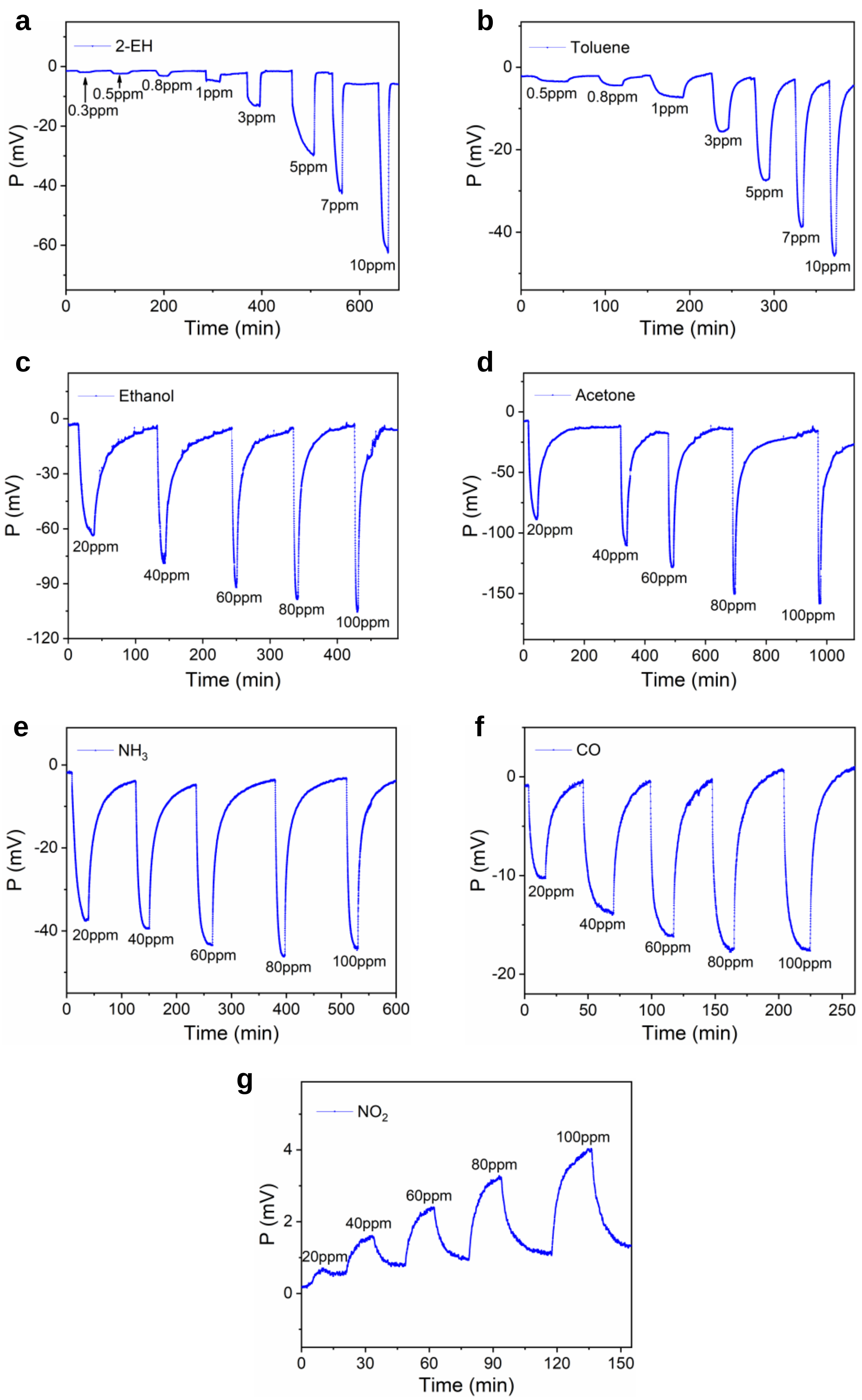

Supplementary Fig. 29. Continuous dynamic potentiometric response curves in (a) 0.3-10ppm 2-EH, (b) 0.5-10ppm toluene, and (c-g) 20-100ppm (c) ethanol, (d) acetone, (e) NH_3, (f) CO, (g) NO_2 for the SnO_2-BSCF sensor at 400 ℃.

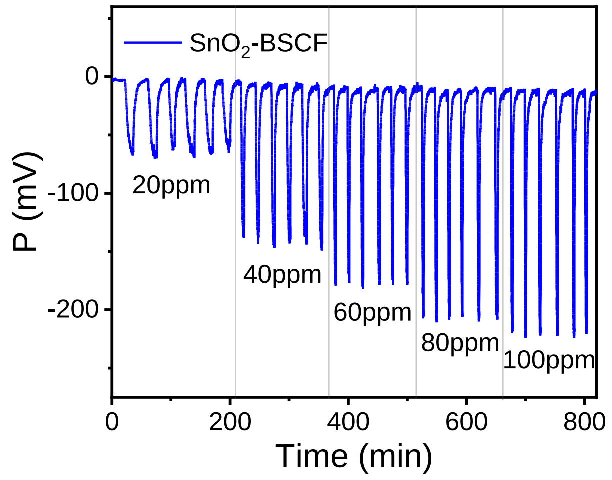

Supplementary Fig. 30. Continuous dynamic potentiometric response curves in 20-100 ppm 2-EH for the SnO_2-BSCF sensor at 400 ℃.

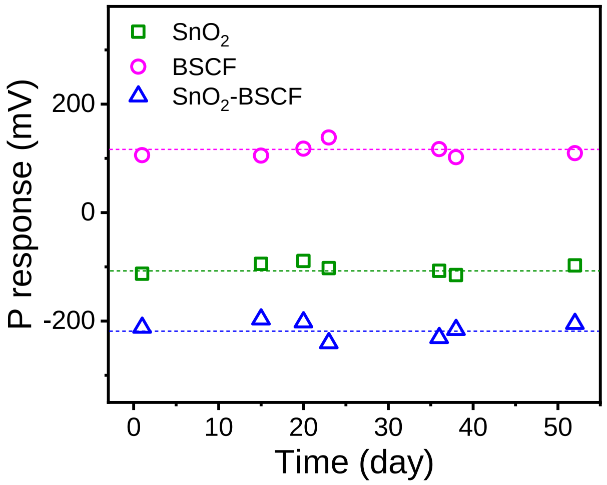

Supplementary Fig. 31. Stability test of the potentiometric response to 100 ppm 2-EH for the SnO_2-BSCF sensor at 400 ℃. Data for SnO_2 and BSCF sensors are also shown for comparison.

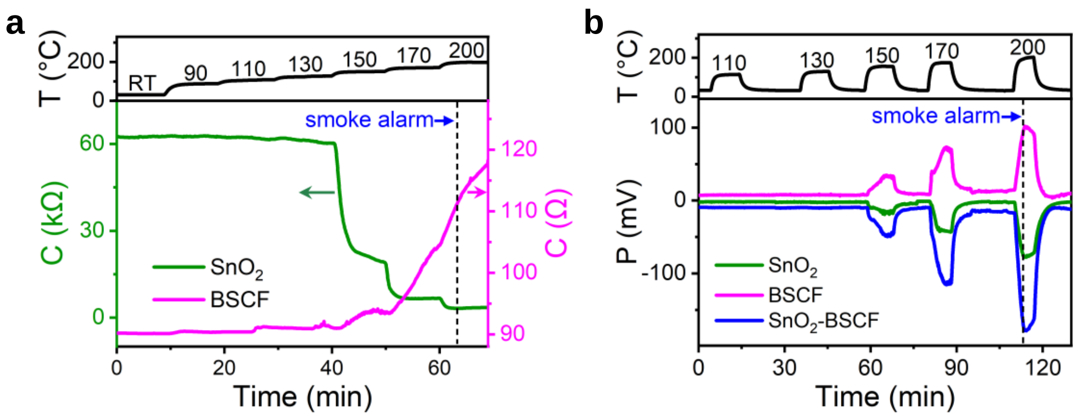

Supplementary Fig. 32. (a) Chemiresistive and (b) potentiometric response curves of SnO_2, BSCF, and SnO_2-BSCF sensors as a function of time at different overheating temperature of Cable 2.

In Supplementary Fig. 32, appreciable resistance decrease for SnO_2 is observed at a cable temperature as low as 110 ℃, while considerable P response starts from ~150 ℃ for both SEs. At 150 ℃, the C response is 3.33 for SnO_2 and 1.04 for BSCF, and a P response as large as -32.06 mV is obtained for SnO_2-BSCF. By contrast, commercial smoke detector does not go off at 170 ℃ or below within a duration of 40 min; it alarms after the cable has been heated to 200 ℃ for a substantial period, when the responses of the C-P sensor are already rather pronounced.

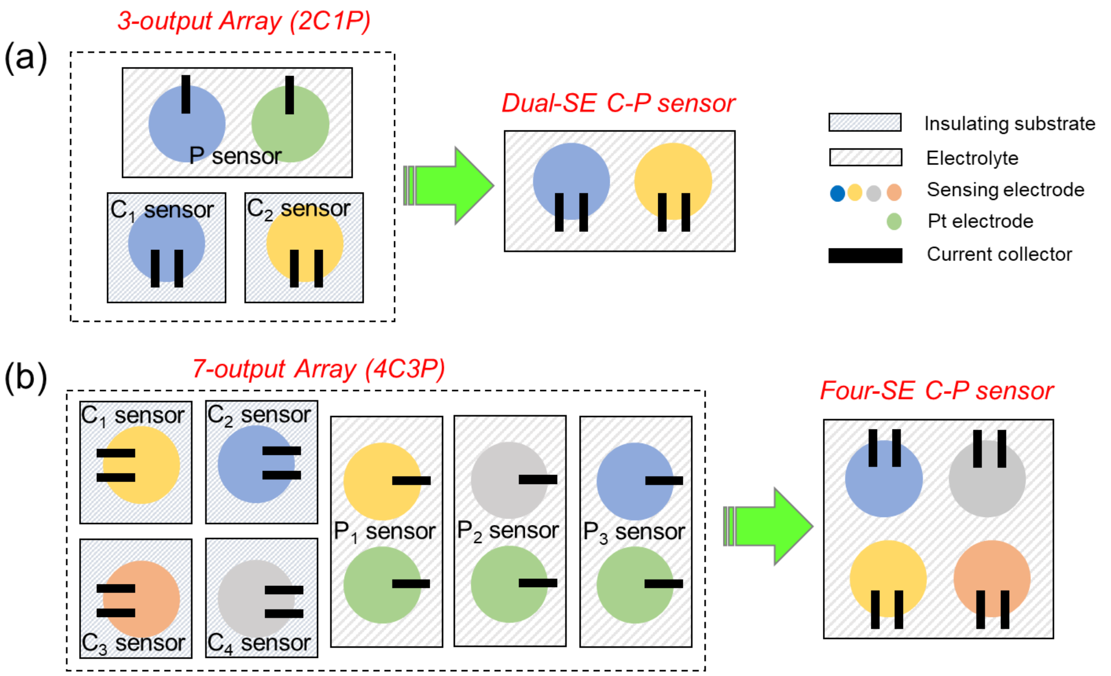

Supplementary Fig. 33. Comparison of conventional arrays and corresponding C-P sensors that output (a) 3 and (b) 7 different independent C/P signals.

The C-P sensors, outputting the same signals as arrays whilst performing better, use less materials (including substrates, Pt, and sensing materials) and are smaller and more compact than arrays. These benefits become more evident when a larger number of SEs are used, which are highly desirable for the portability, miniaturization, and integration, and for large-scale applications in the Internet of Things era.

Supplementary Tables

Supplementary Table 1. State-of-the-art multivariable gas sensors with demonstrated gas discrimination.

Transducers	Change of parameters measured	Response dimension	Number of gases	Concentration range	Ref
chemiresistive, thermoelectric	resistance, Seebeck voltage	2D	6	50-3000 ppm	3
chemiresistive, mass	resistance, acoustic wave resonance frequency	2D	5	10%-100%	4
chemiresistive, mass	resistance, acoustic wave resonance frequency	2D	3	10%-100%	5
chemiresistive, mass	resistance, acoustic wave resonance frequency	2D	2	Saturated vapors	6
chemiresistive, mass	series and parallel resonance frequency	2D	3	Saturated vapors	7
chemiresistive, mass	current, series resonance frequency	2D	4	0-4000 ppm	8
chemiresistive, mass	current, resonant frequency	2D	6	300-1200 ppm	9
chemiresistive, optical	conductance, chemiluminescence	2D	20	0.6%	10
chemiresistive, optical	resistance, surface enhanced Raman scattering	2D	3	0.25-100 ppm	11
chemiresistive, optical	resistance, colorimetric response	2D	8	10-1000 ppm	12
chemiresistive, work function	conductance, surface work function	2D	5	1.5-8 ppm, 0.028%-0.3%	13
chemiresistive, capacitive	conductance, capacitance	2D	2	0.0002-0.02P_0	14
capacitive, optical	capacitance, photodiode output voltage	2D	3	0-0.6P/P_0	15
mass, field effect transistor	resonance frequency, surface potential	2D	5	100-26000 ppm	16
mass, viscoelastic	resonance frequency, resistance	2D	2	(2-10) $\times 10^3$ ppm	17
mass, mechanical	resonance frequency, motional resistance	2D	8	0.190-77.0 mg/L	18
thermoelectric, viscous gas damping	thermal conductivity, resonance frequency	2D	8	0-100%	19
chemiresistive, capacitive, work function	conductance, capacitance, surface work function	3D	2	1ppm, 550 ppm	20
optical	surface photovoltage, photoluminescence	2D	20	15.3-79.2 mg/L	21
impedance	impedance spectra	2D	3	0.044-0.089 P/P_0	22
impedance	impedance spectra	2D	4	0.03-0.15P/P_0	23
impedance	impedance spectra	2D	5	1×10^{-5}-0.2P/P_0	24
impedance	impedance spectra	3D	3	5-30 ppm	25
impedance	impedance spectra	3D	4	100-800 ppm	26
chemiresistive, potentiometric	resistance, open circuit voltage	3D	8	0.3-100 ppm	This work

Supplementary Table 2. Overall recognition accuracy for 6 target gases based on LDA using the sensor arrays with different signals.

Sensor array with different signals	Accuracy (%)
C(SnO_2), P(SnO_2)	76.5
C(LSCF), P(LSCF)	88.2
C(SnO_2), P(LSCF)	73.5
C(LSCF), P(SnO_2)	85.3
C(SnO_2), C(LSCF)	55.9
P(SnO_2), P(LSCF)	88.2
C(SnO_2), C(LSCF), P(SnO_2)	91.2
C(SnO_2), C(LSCF), P(LSCF)	88.2
C(SnO_2), P(SnO_2), P(LSCF)	88.2
C(LSCF), P(SnO_2), P(LSCF)	91.2
**C-P sensor with C(SnO_2), C(LSCF), P(SnO_2-LSCF)**	**91.2**

The accuracy of different signal combinations follows an order of PP>CP>CC, indicating that the P signal is more advantageous in gas recognition than the C one when combined with other C/P signals. The dual-SE C-P sensor with three different outputs, i.e., C(SnO_2), C(LSCF), and P(SnO_2-LSCF), exhibits better discrimination capability than the single-SE one. Moreover, its discrimination performance is equivalent or even superior to that of conventional arrays with three single-output C/P sensors of SnO_2 and LSCF.

Supplementary Table 3. LOD data of P(SnO_2-BSCF) sensor for 7 target gases.

Gas	LOD (ppm)	TWA (ppm) *
2-EH	0.00811	1
Toluene	0.05591	50
Ethanol	1.39	1000
Acetone	0.85	500
NH_3	1.65	20
CO	1.53	20
NO_2	1.28	0.5

*: Occupational Exposure Limits of European Union[27] or OSHA (ethanol)[28].

Supplementary Table 4. Overall recognition accuracy of the SnO_2-BSCF multivariate C-P sensor and different arrays for 7 target gases and humidity.

Sensor array with different signals	Accuracy (%)
C(SnO_2), P(SnO_2)	52.1
C(BSCF), P(BSCF)	84.9
C(SnO_2), P(BSCF)	79.5
C(BSCF), P(SnO_2)	68.5
C(SnO_2), C(BSCF)	65.8
P(SnO_2), P(BSCF)	87.7
C(SnO_2), C(BSCF), P(SnO_2)	72.6
C(SnO_2), C(BSCF), P(BSCF)	94.5
C(SnO_2), P(SnO_2), P(BSCF)	94.5
C(BSCF), P(SnO_2), P(BSCF)	89.0
C(SnO_2), C(BSCF), P(SnO_2), P(BSCF)	95.9
**C-P sensor with C(SnO_2), C(BSCF), P(SnO_2-BSCF)**	**97.3**

The accuracy follows generally an order of PP>CP>CC, which is similar to that for SnO_2-LSCF (Supplementary Table 2), confirming the advantage of the P signal in gas recognition than the C one. The dual-SE C-P sensor with three different outputs, i.e., C(SnO_2), C(BSCF), and P(SnO_2-BSCF), exhibits a high recognition accuracy of 97.3%, outperforming conventional 2-sensor arrays of SnO_2 and BSCF (CC, CP, PP), the 3-sensor one (CCP、CPP), and even the 4-sensor one (CCPP).

Supplementary Table 5. Detailed parameters of the structures and SE sintering conditions for different multivariable sensors.

**Number of SE**	**Sensors**	**SE materials**	**SE sintering temperature**	**Electrode sintering time**
Single SE	SnO_2-Pt, ZnO-Pt, NiO-x-Pt	SnO_2, ZnO, NiO-x	600 ℃	3 h
	LSCF-Pt	LSCF	1000 ℃	3 h
	BSCF-Pt	BSCF	950 ℃	3 h
Dual SEs	SnO_2-BSCF	SnO_2, BSCF	SnO_2-600 ℃, BSCF-950 ℃	3 h
Four SEs	NiO-x1 vs NiO-x2	NiO-0, NiO-0.5, NiO-3, NiO-10	600 ℃	3 h

Supplementary References

1. Patel KN, Deshpande M, Gujarati VP, Pandya S, Sathe V, Chaki S. Structural and optical analysis of Fe doped NiO nanoparticles synthesized by chemical precipitation route. *Materials Research Bulletin* **106**, 187-196 (2018).
2. Qiu Z, Ma Y, Edvinsson T. In operando Raman investigation of Fe doping influence on catalytic NiO intermediates for enhanced overall water splitting. *Nano Energy* **66**, 104118 (2019).
3. Ionescu R. Combined Seebeck and resistive SnO_2 gas sensors, a new selective device. *Sensors and Actuators B: Chemical* **48**, 392-394 (1998).
4. Gao F, Xuan W, Bermak A, Boussaid F, Tsui C-Y, Luo J. Dual transduction on a single sensor for gas identification. *Sensors and Actuators B: Chemical* **278**, 21-27 (2019).
5. Gao F, Boussaid F, Xuan W, Tsui C-Y, Bermak A. Dual transduction surface acoustic wave gas sensor for VOC discrimination. *IEEE Electron Device Letters* **39**, 1920-1923 (2018).
6. Hwang B, Yang J, Lin C. Recognition of alcohol vapor molecules by simultaneous measurements of resistance changes on polypyrrole-based composite thin films and mass changes on a piezoelectric crystal. *Sensors and Actuators B: Chemical* **75**, 67-75 (2001).
7. Leong A, Saha T, Swamy V, Ramakrishnan N. A Langasite Crystal Microbalance Coated with Graphene Oxide-Platinum Nanocomposite as a Volatile Organic Compound Sensor: Detection and Discrimination Characteristics. *Sensors* **20**, 334 (2020).
8. Chen Y, et al. Chemiresistive and gravimetric dual-mode gas sensor toward target recognition and differentiation. *ACS Applied Materials & Interfaces* **8**, 21742-21749 (2016).
9. Yu YY, et al. Volatile organic compounds discrimination based on dual mode detection. *Nanotechnology* **29**, 245502 (2018).
10. Liu D, Liu M, Liu G, Zhang S, Wu Y, Zhang X. Dual-channel sensing of volatile organic compounds with semiconducting nanoparticles. *Analytical chemistry* **82**, 66-68 (2010).
11. Han HJ, et al. Synergistic Integration of Chemo-Resistive and SERS Sensing for Label-Free Multiplex Gas Detection. *Advanced Materials* **33**, 2105199 (2021).
12. Khatib M, et al. Hierarchical Graphene-Dye Bilayers for Multimodal Optoelectronic Sensing and Decoupling of Complex Stimuli. *Advanced Materials Technologies* **8**, 2200920 (2023).
13. Qazi M, Vogt T, Koley G. Two-dimensional signatures for molecular identification. *Applied*

Physics Letters **92**, 103120 (2008).

14. Snow ES, Perkins FK. Capacitance and conductance of single-walled carbon nanotubes in the presence of chemical vapors. *Nano Letters* **5**, 2414-2417 (2005).
15. Jalkanen T, Tuura J, Makila E, Salonen J. Electro-optical porous silicon gas sensor with enhanced selectivity. *Sensors and Actuators B: Chemical* **147**, 100-104 (2010).
16. Di Natale C, Paolesse R, D'Amico A, Lundström I, Lloyd-Spetz A. Multi-transduction of molecular recognition events in metalloporphyrin layers. *Journal of Porphyrins and Phthalocyanines* **13**, 1123-1128 (2009).
17. Holloway A, Nabok A, Thompson M, Ray A, Wilkop T. Impedance analysis of the thickness shear mode resonator for organic vapour sensing. *Sensors and actuators B: Chemical* **99**, 355-360 (2004).
18. Regmi BP, et al. A novel composite film for detection and molecular weight determination of organic vapors. *Journal of Materials Chemistry* **22**, 13732-13741 (2012).
19. Loui A, Sirbuly D, Elhadj S, McCall S, Hart B, Ratto T. Detection and discrimination of pure gases and binary mixtures using a dual-modality microcantilever sensor. *Sensors and Actuators A: Physical* **159**, 58-63 (2010).
20. Nomani MW, et al. Highly sensitive and multidimensional detection of NO_2 using In_2O_3 thin films. *Sensors and Actuators B: Chemical* **160**, 251-259 (2011).
21. Hu J, Jiang XM, Wu L, Xu KL, Hou XD, Lv Y. UV-Induced Surface Photovoltage and Photoluminescence on n-Si/TiO_2/TiO_2:Eu for Dual-Channel Sensing of Volatile Organic Compounds. *Analytical Chemistry* **83**, 6552-6558 (2011).
22. Nagraj N, Slocik JM, Phillips DM, Kelley-Loughnane N, Naik RR, Potyrailo RA. Selective sensing of vapors of similar dielectric constants using peptide-capped gold nanoparticles on individual multivariable transducers. *Analyst* **138**, 4334-4339 (2013).
23. Potyrailo RA, Burns A, Surman C, Lee D, McGinniss E. Multivariable passive RFID vapor sensors: roll-to-roll fabrication on a flexible substrate. *Analyst* **137**, 2777-2781 (2012).
24. Potyrailo RA, Surman C, Go S, Lee Y, Sivavec T, Morris WG. Development of radio-frequency identification sensors based on organic electronic sensing materials for selective detection of toxic vapors. *Journal of Applied Physics* **106**, 124902 (2009).
25. Kaur P, Bagchi S, Gribble D, Pol VG, Bhondekar AP. Impedimetric Chemosensing of Volatile

Organic Compounds Released from Li-Ion Batteries. *ACS Sensors* **7**, 674-683 (2022).

26. Li DS, et al. Virtual sensor array based on MXene for selective detections of VOCs. *Sensors and Actuators B: Chemical* **331**, 129414 (2021).
27. Hunter W, Aresini G, Haigh R, Papadopoulos P, Von der Hude W. Occupational exposure limits for chemicals in the European Union. *Occupational and Environmental Medicine* **54**, 217 (1997).
28. Mahmud MM, et al. A Low-Power Wearable E-Nose System Based on a Capacitive Micromachined Ultrasonic Transducer (CMUT) Array for Indoor VOC Monitoring. *IEEE Sensors Journal* **21**, 19684-19696 (2021).
